# Usability Testing of a Social Media Chatbot for Increasing Physical Activity Behavior

**DOI:** 10.3390/jpm12050828

**Published:** 2022-05-20

**Authors:** Dillys Larbi, Kerstin Denecke, Elia Gabarron

**Affiliations:** 1 Norwegian Centre for E-Health Research, 9019 Tromso, Norway; elia.gabarron@ehealthresearch.no; 2 Department of Clinical Medicine, Faculty of Health Sciences, UiT The Arctic University of Norway, 9037 Tromso, Norway; 3 Institute for Medical Informatics, Bern University of Applied Sciences, 3012 Bern, Switzerland; kerstin.denecke@bfh.ch; 4 Department of Education, ICT and Learning, Østfold University College, 1757 Halden, Norway

**Keywords:** social media, physical activity, chatbot, health, participatory health, usability, conversational agent, behavior change

## Abstract

Digital interventions for increasing physical activity behavior have shown great potential, especially those with social media. Chatbots, also known as conversational agents, have emerged in healthcare in relation to digital interventions and have proven effective in promoting physical activity among adults. The study’s objective is to explore users’ experiences with a social media chatbot. The concept and the prototype development of the social media chatbot MYA were realized in three steps: requirement analysis, concept development, and implementation. MYA’s design includes behavior change techniques effective in increasing physical activity through digital interventions. Participants in a usability study answered a survey with the Chatbot Usability Questionnaire (CUQ), which is comparable to the Systems Usability Scale. The mean CUQ score was below 68, the benchmark for average usability. The highest mean CUQ score was 64.5 for participants who thought MYA could help increase their physical activity behavior. The lowest mean CUQ score was 40.6 for participants aged between 50 and 69 years. Generally, MYA was considered to be welcoming, very easy to use, realistic, engaging, and informative. However, some technical issues were identified. A good and diversified user experience promotes prolonged chatbot use. Addressing identified issues will enhance users’ interaction with MYA.

## 1. Introduction

Decades of research show that physical activity interventions can reduce the risk of chronic conditions such as obesity, heart disease, type 2 diabetes, or depression, among others [1,2], and help reduce healthcare costs [3]. The increasing levels of adult inactivity in recent years highlight a clear challenge with developing interventions capable of engaging the adult population in physical activity [4]. 

There is a positive association between the use of digital technologies as interventions and an increase in physical activity behavior. The use of digital interventions for increasing adult physical activities has shown great improvement and potential [4,5,6,7,8]. In a review by Petersen et al. [9], digital interventions for physical activity incorporating a social media element were more engaging to adults and therefore more effective than those without social media. Chatbots, also known as conversational agents or virtual agents, have emerged in the health sector in relation to digital interventions. The psychological and pedagogical effect of spoken opinions vs. written recommendations is evident, establishing effective relationships. In this sense, computer software programs simulating a human conversation via text or voice have been used to either manage chronic conditions or promote healthy behaviors including physical activity behavior [4,10,11]. 

According to Luo et al. [4], chatbots are effective in promoting physical activity among adults. The popularity of social media platforms and the intuitive nature of chatbots suggest a potentially effective means of promoting physical activity if these tools are combined. One of the benefits of a social media-based chatbot for behavior change is that users are already familiar with communicating with friends via social media platforms on a regular basis. We have developed a prototype of a social media-based chatbot that aims to motivate users to be more physically active, that is, to increase their number of steps per day. The chatbot acts as a friend and contacts users via a social media platform with physical activity-related information mainly for inspiration.

The objective of this study is to explore users’ experiences with the social media chatbot and to assess its potential to change physical activity behavior. User evaluation of digital health interventions at an early stage of the development process is essential to ensure that the resulting tool is acceptable and useful to the target population. For this reason, we focused our study on the assessment of user experiences when using the first version of the social media chatbot. 

## 2. Materials and Methods

### 2.1. System Development and Requirement Analysis

The concept and the prototype development of the social media chatbot were realized in three steps: requirement analysis, concept development, and implementation. Requirements were collected in close collaboration with experts in health informatics and psychology. Additional requirements were obtained by reviewing relevant literature for behavior change strategies to motivate individuals. Further details about the development process can be found in Larbi et al. [12].

A chatbot called MYA, integrated into an existing social media platform (Telegram Messenger), was developed for the following reasons: (1) A user can easily add the chatbot to the Telegram application and start communicating with it; (2) no additional app has to be installed; (3) since most people can interact via a social media messenger, interacting with the chatbot will be intuitive and understandable. 

### 2.2. Behavior Change Techniques and Functionalities

MYA’s design includes behavior change techniques that have been proven to be effective in increasing physical activity behavior in digital interventions [7]: goals and planning; feedback and monitoring; social support (unspecified); associations (prompts/cues); and reward and threat (social reward) [13]. In more detail, MYA allows the user to set a personal step goal and review this goal. The chatbot is designed to compare the user’s current number of steps (simulated number of steps in this prototype) with the set goal and inform the user of discrepancies between current behavior and goal. In this context, MYA gives feedback on the user’s behavior. The chatbot also encourages users (if they achieve their goals or are about to achieve them), and it sends prompts, reminding the user about his/her commitment to increasing physical activity. 

MYA is a rule-based chatbot developed using FlowXo [14], which is a platform to create chatbot flows. Table 1 shows the nine conversation flows implemented in MYA. With the trigger word “menu”, a flow is activated where the user can choose whether he/she wants to set goals or challenges, check his/her current step count, or hear a fun fact about exercising. When a user sends MYA a message, a distinction is made between the “First Encounter” when MYA is used for the very first time and a “Further Encounter” flow for each subsequent use. MYA encourages the user to complete his/her specified number of steps per day by sending motivational messages in the chat. 

The entire conversation flow of MYA was designed using the Business Process Model and Notation standard (BPMN). The BPMN models were then translated into conversation streams within FlowXo. To enable personalization of step goals and challenges, Google Sheets was used to collect and store unencrypted non-personal data during conversations with MYA, including current challenge, daily step goal, and first encounter status.

### 2.3. Preliminary Study on MYA’s Usability

We carried out a study to get feedback on the usability and acceptability of the social media chatbot MYA and to identify issues for improvement. Holmes et al. [15] proposed 26 as a reliable number of participants for studies on chatbot usability. Colleagues of the co-authors aged 18 years and above were invited via a link on social media or email to participate in the study. Study participants had the option to use either the mobile or desktop version of the Telegram application. The participants interacted with the chatbot at their convenience, and then answered a survey that included the Chatbot Usability Questionnaire (CUQ), see Table A1 in Appendix A. 

The CUQ is a chatbot-specific usability questionnaire that is comparable to the Systems Usability Scale (SUS)—a commonly used tool for assessing usability that has a benchmark score of 68 out of a total score of 100 [15]. The 16 CUQ items are ranked out of five, the scores are calculated out of 160 and then normalized to 100. The CUQ assesses aspects related to a chatbot’s personality, onboarding, user experience, and error handling. Using SUS is not recommended for usability testing of conversation-driven systems since they exploit other design principles [15]. A CUQ Calculation Tool—a Microsoft Excel spreadsheet—is available for the easy calculation of CUQ scores for each participant, the mean CUQ score, and the median score [16]. Further details about the usability study are published in Larbi et al. [12].

The CUQ scores were further analyzed using Microsoft Excel and SPSS (version 25; IBM Corp) to create graphs and group statistics. The participants’ gender and age groups were analyzed using Crosstabulation. Bar charts were used to display the participants’ ratings of the positive and negative aspects of the CUQ, and a scatter plot was used to display the participants’ age groups and CUQ scores. NVivo 12 Pro was used to conduct an inductive thematic analysis of participants’ open-ended answers. Each participant’s feedback was read through thoroughly and coded. The generated codes were then categorized into themes and/or subthemes.

### 2.4. Ethics

No personal data were collected for this study. All data were treated confidentially and only used for this usability study. This study was approved by the Institutional Review Board Cantonal Ethics Committee in Bern (BASEC-Nr: Req-2021-00244).

## 3. Results

### 3.1. Participant Characteristics

The survey was answered by 30 adult volunteers between 17 and 26 March 2021. Nine of the 30 study participants were aged between 18 and 29 years, 18 participants were aged between 30 and 49 years, and 3 participants were aged between 50 and 69 years. The self-reported gender and age group of the respondents are listed in Table 2. 

Of the 30 participants, 63.3% (19/30) interacted with MYA for between 5 to 15 min, 16.7% (5/30) had a 15 to 30 min interaction, 13.3% (4/30) interacted for less than 5 min, and 6.7% (2/30) interacted with the chatbot for more than 60 min.

### 3.2. Average Ranking of Chatbot Usability Questionnaire 

**Figure 1:**
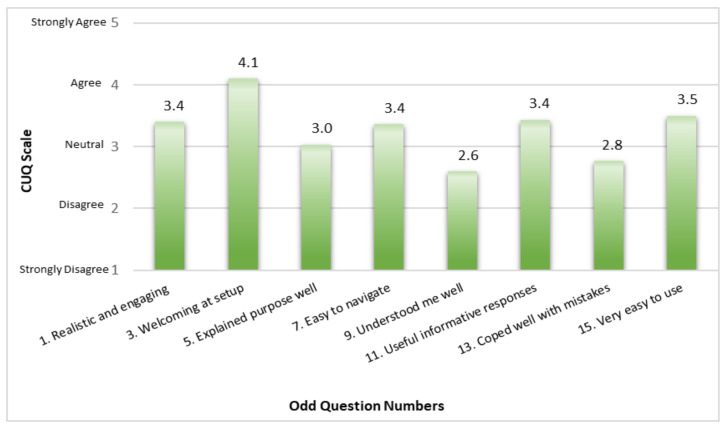
Average ranking for the positive aspects of MYA’s usability.

The odd question numbers of the CUQ have statements that relate to the positive aspects of the chatbot. On a scale of 1—Strongly Disagree to 5—Strongly Agree to the positive statements about MYA’s usability, Question 3, which states ‘The chatbot was welcoming during initial setup’ had the highest average ranking of 4.1 corresponding to Agree. The lowest average ranking was 2.6 for Question 9 which states ‘The chatbot understood me well’ (See Figure 1).

**Figure 2:**
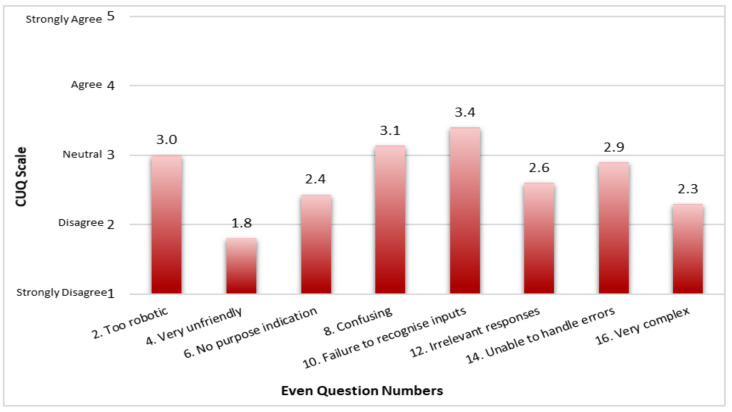
Average ranking for the negative aspects of MYA’s usability.

In Figure 2, the average ranking on a scale of 1—Strongly Disagree to 5—Strongly Agree of the CUQ even question numbers with statements related to the negative aspects of the chatbot are shown. Question 10, which states ‘The chatbot failed to recognize a lot of my inputs’ had the highest average ranking of 3.4. With an average ranking of 1.8, Question 4, which states ‘The chatbot seemed very unfriendly’ had the lowest ranking.

### 3.3. Usability Study Results (According to CUQ Calculator)

**Figure 3:**
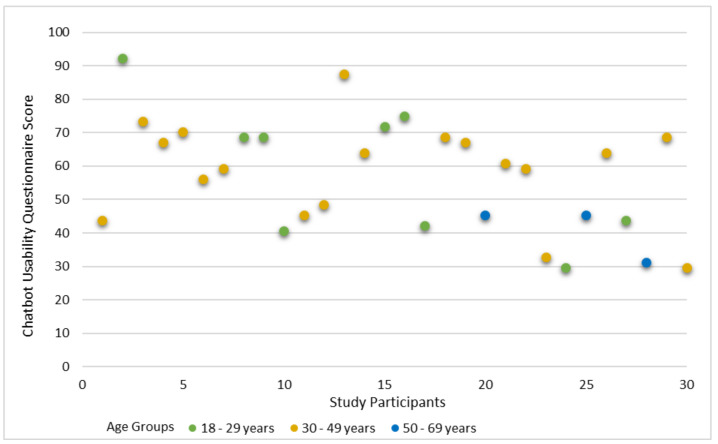
Chatbot Usability Questionnaire (CUQ) Scores for MYA.

In Figure 3, the chatbot usability scores for MYA, the prototype of a physical activity social media chatbot by each participant are illustrated. The highest score was 92.2 and the lowest was 29.7. The mean score was 57.4 ± 16.7 and the median was 60.2. Compared with the benchmark score of 68, the usability of MYA is below average.

Female participants’ median CUQ score was 60.9, and male respondents’ CUQ median was 56.3 (See Table 3). Participants aged 18–29 years reported the highest usability CUQ scores, with a median of 68.8; participants aged 50–69 years had the lowest CUQ scores, with a median of 45.3 (See Table 3).

Of the 30 study participants, 9 used the Telegram desktop app and 21 used the Telegram mobile app. A total of 12 out of the 21 Telegram mobile app users used an Android phone, 7 used an iPhone, and 2 did not specify the type of phone used to chat with MYA. Participants who used the Telegram desktop app had a mean CUQ score of 52.6 and a median score of 43.8 (range 29.7–92.2). 

In general, participants who used the Telegram mobile app had a mean CUQ score of 59.5 and a median score of 64.1 (range 29.7–87.5). Regarding the type of phone, participants who used an Android phone to interact with MYA had a mean CUQ score of 61.5; and iPhone users had a mean CUQ score of 56.9 (see Table 3).

### 3.4. Additional Feedback from Study Participants

Three themes emerged from the analysis of the feedback from the participants. These included:

- Identified issues: “*The only thing was that the app got stuck at times, and it wasn’t clear how to proceed or if this behavior was intended*”
- Preferred chatbot features: “*The random challenge is my favorite feature because it really distinguishes this bot from fitness trackers, and motivates me to do some activity*”
- Suggestions for improvements: “A weekly activity challenge would be interesting, like a schedule with the desired level”, and “*More inputs so that it can talk about everyday subjects like weather and answer some questions*”.

Additionally, some subthemes were identified. A detailed analysis of the comments from the participants is given in Table A2 in Appendix A. 

## 4. Discussion

In this study, we aimed to explore users’ experiences with the physical activity social media chatbot, including identified usability issues. The mean Chabot Usability Questionnaire score was below 68, the benchmark for average usability. The highest mean CUQ score was 64.5, recorded for participants who thought MYA could help increase their physical activity behavior. The lowest mean CUQ score was 40.6 for participants aged between 50 and 69 years.

### 4.1. Social Media Chatbot Features

The results show that the social media chatbot MYA still has potential for improvement: the clarity of the chatbot’s comments and its communication skills should be extended and error handling has to be integrated (i.e., dealing with unexpected user input). In its current prototype stage, MYA’s conversation capabilities are very limited. Extending the small talk functionality and including more variety in the motivational comments or features would be required to ensure user acceptance [17]. Unlike artificial intelligence (AI) chatbot modules that may invoke hesitancy among potential users [18], MYA is a non-AI-based social media chatbot module that interacts with users as a friend and can therefore be effective in increasing and sustaining physical activity among users. It has also been suggested that social rewards that entail active peer-on-peer interactions, such as a chatbot interaction, are effective for sustaining habits [19]. 

Integrating MYA into a social media messenger instead of a stand-alone version of the chatbot also has limitations. Data privacy and data security cannot be guaranteed; the Telegram messenger has—similar to other social media messengers—been criticized with respect to data security [20]. In a stand-alone application, this could be avoided. However, the user would have to install an app which could have negative effects on acceptance. Even though MYA is not supposed to store the real name of a user, users might enter their real names or even a unique username that could make them identifiable.

### 4.2. Can a Social Media Chatbot Help Increase Physical Activity?

In a brief review conducted by Zhang et al. [21] that involved 7 studies on chatbot-based behavior interventions for physical activity and diet, it was found that chatbots can be effective in changing the activity behavior of users. Users of these chatbots, among other things, increased their step-goal achievements [22], physical activity [23,24], and weight loss [25].

In our study, we had mixed opinions on MYA’s potential to impact an individual’s activity behavior, which might be due to the early prototype status of the chatbot that was tested. Furthermore, the maximum duration of participants’ interaction with MYA was 60 min, which limits the chatbot experience and therefore participants’ ability to determine the effect of using it. Our chatbot is still under development so there were few and/or limited functionalities, for example, the integration with an activity tracker was simulated at the time of this study. Some of the study participants realized this and it might have impacted their perception of the chatbot. In addition, the communication skills of MYA were restricted. Another usability and acceptability study will be carried out before testing its efficacy in a clinical trial.

### 4.3. Study Limitations

This study has some limitations. The chatbot did not have a step-counter integrated when the usability test was carried out; the number of steps was randomly generated, which was not appreciated by some participants as it did not reflect the effort made that day. However, we believe it is important to run a usability test at an early stage of system development to ensure a well-accepted system is developed, and in this way, the time spent developing the software is maximized.

The anonymous online survey involved volunteers, mostly students and researchers in the field of digital health or computer science. Therefore, the findings of this study might not be comparable to the general population, nor to other social media chatbots for increasing physical activity behavior. Our study does not provide much insight into the functionality and utility of the chatbot as the focus of the study was on the chatbot prototype’s usability.

## 5. Conclusions

In this paper, we introduced MYA, a social-media-based chatbot for behavior change. Our study indicates that the social media chatbot MYA is welcoming, very easy to use, has a realistic and engaging personality, and provides useful, appropriate, and informative responses. However, some technical issues that need to be fixed were identified and suggestions for improvement were also made.

Further research on the use of chatbots for increasing physical activity could explore the impact of integrating event databases or gadgets and including additional or different behavior change techniques. In addition, future research should investigate the role of different functionalities and the utility of a social media chatbot for increasing physical activity behavior.

It is only when the user experience is good and diversified that the chatbot will be used for a longer period. By integrating the suggested functionalities, we will be able to achieve a wide variation in the way future users interact with MYA.

## Figures and Tables

**Table 1:** Implemented conversation flows of MYA.

Conversation Flow	Description
First encounter	Started only the first time MYA is used. Collects basic information on the user and explains the usage of the chatbot. A daily step goal is specified.
Further encounter	Greeting for any other than the first encounter. MYA asks the user about his well-being and tries to encourage the user.
Menu	Offers access to the four functions: goals, challenges, steps, and facts.
Goals	Allows the user to specify a goal for long-time encouragement.
Challenges	Out of a set of user-tailored challenges, one is selected.
Steps today	Checks the number of steps (simulated step count). This function compares the set goal with current number of steps. If the step goal is not achieved, MYA encourages the user to take more steps.
Facts	Presentation of a randomly selected fact on health and activity behavior.
Chatting	Allows out-of-topic chatting with the bot. Current version of MYA is not designed to start out-of-topic discussions.
Help	Provides help on the various functions.

**Table 2:** Characteristics of the survey respondents (*n* = 30).

Age Group	Gender		
	Female	Male	Total
18–29 years	3 (10%)	6 (20%)	9 (30%)
30–49 years	12 (40%)	6 (20%)	18 (60%)
50–69 years	0	3 (10%)	3 (10%)
Total	15 (50%)	15 (50%)	30 (100%)

**Table 3:** Chatbot Usability Questionnaire (CUQ) Scores for study participants (*n* = 30) according to participant characteristics.

Participant Characteristic	Mean CUQ Score	Median CUQ	Lowest Score	Highest Score
**Gender**				
Female	59.9 ± 18.06	60.9	29.7	92.2
Male	54.9 ± 15.5	56.3	29.7	75.0
**Age Group**				
18 and 29 years	59.2 ± 20.7	68.8	29.7	92.2
30 and 49 years	59.3 ± 14.6	62.5	29.7	87.5
50–69 years	40.6 ± 8.1	45.3	31.3	45.3
**MYA’s ability to increase physical activity behavior**				
Maybe	57.9 ± 16.2	60.2	29.7	92.2
No	49.1 ± 15.5	43.8	31.3	71.9
Yes	64.5 ± 17.7	68.8	29.7	87.5
**Mode of Interaction**				
Telegram desktop app	52.6 ± 21.1	43.8	29.7	92.2
Telegram mobile app	59.5 ± 14.6	64.1	29.7	87.5
Android phone	61.5 ± 14.8	64.1	29.7	87.5
iPhone	56.9 ± 15.7	60.9	31.3	73.4

## Data Availability

Further details from the usability questionnaire can be obtained from the corresponding author (dillys.larbi@ehealthresearch.no).

## Appendix A

**Table A1:** MYA Usability Testing Survey.

1. What is your gender? ☐ Female ☐ Male ☐ Diverse ☐ Prefer not to say					
2. What is your age group? ☐ 18–29 years ☐ 30–49 years ☐ 50 – 69 years ☐ ≥70 years					
3. How often do you do physical activity? ☐ Less than 30 min per day ☐ More than 30 min per day ☐ Less than 30 min per week ☐ More than 30 min per week ☐ Monthly ☐ Never ☐ Other: 4. How did you interact with MYA? ☐ Telegram desktop app ☐ Telegram mobile app (Android) ☐ Telegram mobile app (iPhone) ☐ Other:					
5. Do you think MYA could help you in increasing your physical activity/change your current activity behavior? ☐ Yes ☐ No ☐ Maybe ☐ Other: 6. For approximately how long did you interact with MYA? ☐ Less than 5 min ☐ 5–15 min ☐ 15–30 min ☐ 30–60 min ☐ More than 60 min ☐ Other: 7. **Chatbot Usability Questionnaire** (Holmes et al. 2019)					
	1 -Strongly Disagree	2 -Disagree	3 -Neutral	4 -Agree	5 -Strongly Agree
Q1 The chatbot’s personality was realistic and engaging	☐	☐	☐	☐	☐
Q2 The chatbot seemed too robotic	☐	☐	☐	☐	☐
Q3 The chatbot was welcoming during initial setup	☐	☐	☐	☐	☐
Q4 The chatbot seemed very unfriendly	☐	☐	☐	☐	☐
Q5 The chatbot explained its scope and purpose well	☐	☐	☐	☐	☐
Q6 The chatbot gave no indication as to its purpose	☐	☐	☐	☐	☐
Q7 The chatbot was easy to navigate	☐	☐	☐	☐	☐
Q8 It would be easy to get confused when using the chatbot	☐	☐	☐	☐	☐
Q9 The chatbot understood me well	☐	☐	☐	☐	☐
Q10 The chatbot failed to recognise a lot of my input	☐	☐	☐	☐	☐
Q11 Chatbot responses were useful, appropriate, and informative	☐	☐	☐	☐	☐
Q12 Chatbot responses were not relevant	☐	☐	☐	☐	☐
Q13 The chatbot coped well with any errors or mistakes	☐	☐	☐	☐	☐
Q14 The chatbot seemed unable to handle any errors	☐	☐	☐	☐	☐
Q15 The chatbot was very easy to use	☐	☐	☐	☐	☐
Q16 The chatbot was very complex	☐	☐	☐	☐	☐
8. Any other comments (including suggestions for improvement)?					

**Table A2:** Identified themes from the comments of respondents of the chatbot usability testing survey.

Theme (Subtheme)	Examples of Statements
Identified Issues	
Interaction Difficulties	Many reverse-coded questions—A bit difficult to answer:)
	If I type in ‘challenge’ in a layer where it fits, but apparently not to the Chatbot, it is overwhelmed
Incomplete app design	It’s very inaccurate when it comes to counting steps
	The chatbot makes an “unfinished” impression, e.g., the menu below is not always visible and sometimes it is shown with icons and sometimes with / text
Spelling errors	Some spelling mistakes (e.g., writte (write), smartes (smartest), reapeat (repeat), etc.
	A lot of spelling errors (reapeat instead of repeat, smartes instead of smartest, etc)
Unresponsive/frozen app	The only thing was that the app got stuck at times, and it wasn’t clear how to proceed or if this behavior was intended
	If you want to create a new goal and have him help you with it, he hangs himself up
Preferred Chatbot features	
Challenge feature	The random challenge is my favorite feature because it really distinguishes this bot from fitness trackers, and motivates me to do some activity
Goal feature	I also like the functionality for checking the user-defined goals
Suggestions for Improvement	
Challenge related suggestions	
Avoid repeating challenge	When the user does not accept the proposed challenge and asks for a different one, the chatbot should avoid suggesting the same one again
Personalize challenges	It may be nice for the user to be able to personalize the types of challenges (e.g., in the one-time welcome phase, ask the user to select the types of exercise he/she is never going to accept, that can be excluded from the suggestions)
Weekly activity challenge	A weekly activity challenge would be interesting, like a schedule with the desired level.
Interaction related suggestions	
More facts and input	The idea of a chatbot is cool, but it would need to be connected to services and give more inputs. For example, find activities near you that you can do and suggest them
	The *chat* is too fixed. Would have been much better if it could take varied answers.
	More facts should be linked to the chatbot.
	More inputs so that that it can talk about everyday subjects like weather n answer some questions
	It could be useful if it answered at least a generic sentence, or if it prompted the initial menu again.
More empathy and motivation	Finally, she congratulates that I did 2294 steps even though I did not set a goal and she does not motivate me to set one. I would prefer if she could be more empathic and encourage me to set a goal.
Options always available	The second time I tried the chatbot was a bit weird. The chatbot was in a kind of “stand by” mode, in order to discuss goals/steps/etc... again, you need to remember to type “/menu”. Perhaps the different options should be shown all the time.
	Entries should be checked for their meaningfulness. If menu suggestions are made, then these should also work
